# Exploration of the barriers, enablers and experiences of opioid management for chronic non-cancer pain (CNCP) in the general practice setting within the United Kingdom: A meta-synthesis

**DOI:** 10.1177/20494637251393241

**Published:** 2025-10-27

**Authors:** Wendy Chau, Jacquie Ridge

**Affiliations:** 1 6087 Faculty of Health, Education and Society, University of Northampton, UK

**Keywords:** chronic non-cancer pain, opioids, barriers, enablers, primary care

## Abstract

**Background:**

There is insufficient evidence to indicate using opioids in the management of chronic non-cancer pain (CNCP), yet ongoing prescribing is prevalent and remains a global public health matter. Inappropriate long-term prescribing of opioids is associated with side effects and adverse events. This article explores the significant barriers and enablers during opioid management indicated for CNCP within general practice, in the United Kingdom, and proposes recommendations to optimise practice.

**Methods:**

A systematic literature review of the databases MEDLINE, EMBASE, CINAHL, Web of Science and Science Direct were searched. Titles, abstracts and full texts were screened against inclusion and exclusion criteria. Papers were evaluated using the Critical Appraisal Skills Programme qualitative appraisal tool.

**Results:**

From 1027 citations, 6 papers were included (*n* practitioners = 168 and *n* patients = 52). Four key themes were identified: three barriers and one enabler. Barriers: general practice healthcare model constraints, relationships in primary care and attitudes towards CNCP management. Enabler: multidisciplinary team set up.

**Conclusion:**

A change in culture from both service providers and service users is required to fully embrace the multidisciplinary team observed in general practice. Movement away from the traditional model of doctor led management needs to occur. Future policies need to prioritise reducing the long waiting times observed for specialist pain services. Non-pharmacological opportunities and services should also be developed to support patients.

## Introduction

Chronic non-cancer pain (CNCP) is defined as pain lasting for more than 3 months that is unrelated to a cancer diagnosis.^1^ The International Association for the Study of Pain describes pain as ‘an unpleasant sensory and emotional experience associated with actual or potential tissue damage’.^2^ There is insufficient evidence to justify the use of opioids in the management of CNCP. Despite this, in the United Kingdom (UK), the prescribing of opioids for CNCP has progressively increased in the last 20 years.^3^ Within the community setting in England, opioid prescribing for a minimum of 3 months has increased by 54% between 2015 and 2021.^4^

Inappropriate long-term prescribing of opioids is associated with side effects including constipation, dizziness, sedation, nausea, dependence and addiction.^5^ Moreover, although rare, significant immune and endocrine system disturbances, including osteoporosis, have been observed.^6,7^ Adverse events including falls, hospital admissions and opioid related death are also prevalent.^8^ Opioids contribute to nearly half of drug-related deaths. In 2022, there were 2261 opioid related deaths within England and Wales, 1.9% higher from 2021.^9^

Evidence supports the use of opioids in the management of acute pain, following a surgical intervention, cancer related pain and end of life pain. In England the bulk of repeat opioid prescribing occurs from primary care settings, contributing to high opioid prescribing rates.^10^ English prescribing data between 2017 and 2018 showed that 5.6 million people, 13% of the population, had dispensed a prescription for an opioid medicine.^11^

A significant financial investment supports the enhancement of the primary care structure.^12^ Formation of Primary Care Networks (PCNs) in 2019 has seen a multidisciplinary team (MDT) approach at primary care level, increasing the presence of pharmacy professionals, occupational therapists and physiotherapists to name a few. PCN’s aim to increase access to healthcare, support capacity and meet the needs of the community.^13^

This qualitative meta-synthesis aims to explore the current barriers and enablers encountered in the management of opioids for CNCP within the UK general practice setting. This review addresses the experiences of both clinicians and patients’ perspectives. Additionally, proposing recommendations to optimise opioids prescribed for CNCP.

## Methods

This study was conducted in the form of a meta-synthesis qualitative systematic review. Reporting followed Preferred Reporting Items for Systematic review and Meta-Analysis (PRISMA) guidelines.

An initial search was conducted on EMBASE. This identified the search terms reflecting the language, synonyms and medical terms related to UK practice (Table 1).

**Table 1.** Search terms and synonyms.

Search term	Synonym (OR–Boolean)
Opioid	-
Chronic non-cancer pain	Non-cancer pain, pain
General practice	Family practice, primary care
Barriers	Concerns, limitations, constraints
Enablers	Facilitators, promoter
Deprescribing	Medicines optimisation

A systematic search was conducted by one reviewer using the published literature databases MEDLINE, EMBASE, CINAHL, Web of Science and Science Direct between 1^st^ December 2023 and 31^st^ January 2024.

Studies identified were first reviewed by title and abstract. Potentially eligible studies were then reviewed completely and identified as eligible or excluded as per Table 2.

**Table 2.** Inclusion and exclusion criteria.

Inclusion	Exclusion	Rationale
Chronic non-cancer pain	Palliative care, cancer pain and neuropathic pain	Isolated CNCP
Opioids	Non-steroidal anti-inflammatory drugs, gabapentinoids and benzodiazepines	Isolated opioid class of drugs
UK practice	Non-UK practice	Isolated UK practice
Published in English language	Non-English language publications	Ensured that studies could be fully understood and avoid language barriers
Primary care	Secondary care, tertiary care	Isolated primary care general practice settings
Qualitative data analysis methods	Quantitative methods and mix-methods	To ensure deep exploration of experiences and insights supporting theme identification
Full research papers	Conference abstracts and presentation	To include text with full details and exclude limited detail within the text that may be present within abstracts and presentations

The Critical Appraisal Skills Programme (CASP) qualitative appraisal tool was developed to support with appraising health-related research. CASP is endorsed by Cochrane and the World Health Organization and is used for health and social care qualitative synthesis.^14,15^ It uses a simple method of 10 questions to evaluate and critique on significance of results.^16^ Each study was critically appraised using CASP.

Braun et al.’s^17^ thematic analysis was employed from the start of synthesis and throughout the review. This analysis enabled the key topics to be identified and supported with ‘exploring, interpreting and reporting relevant patterns of meaning’.^17^ Six phases were used to analyse the data including coding and theme generation. See Appendix 1.

### Ethical approval and risk of bias

Ethical approval was not required. A sole researcher working in primary care carried out the review and unconscious notion of bias must be acknowledged. Reflexivity and the individuals’ preconceptions that impact on the decisions made during research influences subsequent credibility. Positively, an understanding of the general practice processes provides a valuable insider perspective. However, personal experience of supporting with opioid management can result in interpretivism. Therefore, as the sole researcher, it is important to aim for transparency. Rigour supports the management of any bias and enables auditability. This was maintained through adherence to strict inclusion and exclusion criteria as previously discussed in Table 2.

## Results

The search retrieved 1027 papers after removing duplicates, and all papers had their titles and abstracts screened. Following screening of titles and full text, 6 papers were eligible to be included in analysis (Figure 1).

**Figure 1.**
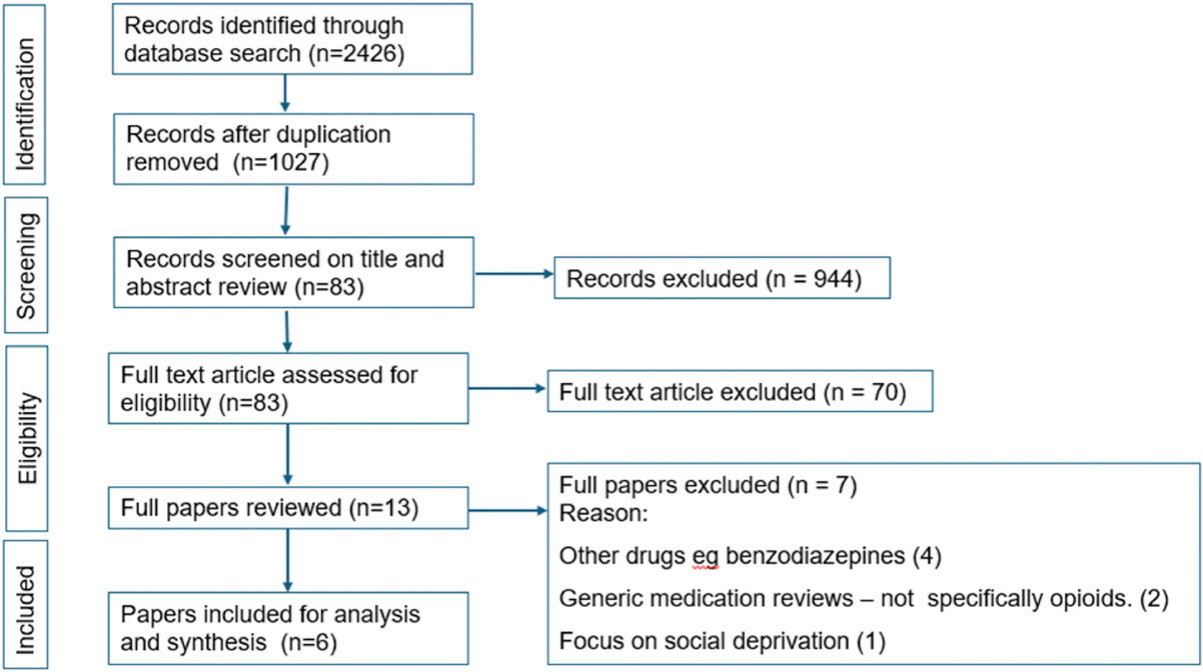
PRISMA flow chart illustrating the results of search strategy.

### Study characteristics

The 6 studies included a total of 220 participants; 168 clinicians and 52 patients. Studies used focus groups,^18–20^ interviews^18–22^ and written surveys^23^ to collect data. One paper addressed the patient perspective.^19^ One paper addressed both patient and general practitioner (GP) perspectives,^20^ and another looked at advance nurse practitioner (ANP) experiences.^21^ Three papers reviewed perspectives of GPs only.^18,22,23^ (Table 3).

**Table 3.** Characteristics of included studies.

Study	Patient/health professional	Data collection methods	Relation between researcher and participants	Second researcher for coding and theme development
Seamark et al.^18^	GPs (*n* = 29)	Focus groups	Unknown	No
		Interviews		
Blake et al. (1)^19^	Patient (*n* = 10)	Focus groups	Patient researcher	No
		Interviews		
McCorrie et al.^20^	Patients (*n* = 23) and GPs	Focus groups	Unknown	Yes
	(*n* = 15)	Interviews		
McCann et al.^21^	ANP (*n* = 10)	Interviews	Unknown	Yes
Blake et al. (2)^23^	GPs (*n* = 94)	Written survey	Unknown	No
Gill et al.^22^	GPs (*n* = 20)	Interviews	Researcher known to participants	Yes

### Quality assessment

All studies demonstrated rigorous design, recruitment, data collection and analysis methods, therefore presenting moderate quality. Blake et al. (1)^19^ and McCorrie et al.^20^ both studied patient perspectives. Selection bias may be present as GPs supported with patient selection during invitation stage. Patients with valuable experiences may not have been invited to participate. Four studies^18,20,21,23^ do not clearly document the relationship between the researchers and the participants.

Gill et al.^22^ utilised an interviewer known to the participants and Blake et al. (1)^19^ utilised a patient researcher, which presents additional challenges including social desirability bias. However, both studies document a robust data analysis process. Gill et al.^22^ utilised multiple researchers to support with analysis and refining themes.

Although a small study population of 10 participants, McCann et al.^21^ add value as all participants were ANPs. This could be both a limitation and strength. The limitation presenting as a lack of exploration of the views of other allied health professionals (AHPs) and the strength as an insight into provision of AHP managing CNCP. McCorrie et al.,^20^ McCann et al.^21^ and Gill et al.^22^ utilised second researchers to support the reliability of coding and theme development, demonstrating robust methodologies (Table 3).

### Synthesis

Results indicate four main themes and subsequent subthemes (Table 4).

**Table 4.** Themes and subthemes identified and corresponding papers.

Theme	Papers supporting theme	Subtheme	Papers supporting subtheme
General practice healthcare model constraints (barrier)	Blake et al. (1)^19^	Service challenges	McCorrie et al.^20^
	McCorrie et al.^20^		McCann et al.^21^
	McCann et al.^21^		Gill et al.^22^
	Gill et al.^22^	Appointment times	Blake et al. (1)^19^
			McCorrie et al.^20^
			Gill et al.^22^
		No alternatives to opioid	McCorrie et al.^20^
			McCann et al.^21^
			Gill et al.^22^
Relationships in primary care (barrier)	Seamark et al.^18^	Patient expectations	Blake et al. (1)^19^
	Blake et al. (1)^19^		McCorrie et al.^20^
	McCorrie et al.^20^		McCann et al.^21^
	McCann et al.^21^		Gill et al.^22^
	Blake et al. (2)^23^	Liability	Seamark et al.^18^
	Gill et al.^22^		Blake et al. (2)^23^
			Gill et al.^22^
Attitudes towards CNCP (barrier)	Seamark et al.^18^	CNCP is difficult to manage	Seamark et al.^18^
	McCorrie et al.^20^		McCorrie et al.^20^
	McCann et al.^21^		McCann et al.^21^
	Blake et al. (2)^23^		Blake et al. (2)^23^
	Gill et al.^22^		Gill et al.^22^
		Fear of opioids vs fear of pain	McCorrie et al.^20^
			Blake et al. (2)^23^
			Gill et al.^22^
Multidisciplinary team set up (enabler)	McCann et al.^21^	Clinical pharmacist support	McCann et al.^21^
	Gill et al.^22^		Gill et al.^22^

#### Theme 1: General practice healthcare model constraints (barrier)

##### Service challenges

Patients and clinicians expressed a lack of continuity of care. Multiple health professionals managing a single patient resulted in a lack of consistency and inability to fully understand a patient’s beliefs and behaviours.^20–22^ Gill et al.^22^ stressed that inputs from different clinicians resulted in challenges for patient management plans to be followed correctly. On the other hand, intervention from a different clinician provides a new insight and opportunity to suggest alternative strategies for management. However, further research is required to support this.

McCorrie et al.^20^ study was carried out in the north of England where prescribing rates of opioids are at higher levels than in other parts of the country. Increased socioeconomic deprivation has been shown to be associated with increased opioid prescribing.^24^ Higher opioid prescribing levels exhibit higher demands and pressures on services; therefore, expressions are more informative and comprehensive. In contrast, Gill et al.,^22^ and McCann et al.^21^ studies were carried out in other geographical areas representing different service pressures for CNCP management. Despite this variation, the same concerns regarding lack of continuity of care were observed.

High workloads and staff shortages also resulted in opioids being prescribed without a formal medication review. Long waiting times following referral to local chronic pain services contributed to opioid continuation, and ongoing prescribing becomes the normal behaviour.^20–22^

##### Appointment times

Patients expressed that GPs did not have the time to listen and address their concerns.^19^ Expectedly, clinicians also highlighted that the lack of consultation time resulted in the inability to address all their patient’s needs.^20,22^ GPs expressed that time pressures contributed to repeat prescribing of opioids rather than making a shared management plan or exploring other ways to support with pain management.^20,22^ These three studies were carried out within different years over the past decade, demonstrating a continuing theme of concern regarding appointment time from both service providers and users.

##### No alternatives to opioids

A lack of alternatives to opioids contributed to ongoing and longer-term prescribing of opioid medications.^20–22^ Although clinicians may be motivated to support with inappropriate prescribing, findings suggest that clinicians found it was challenging with lack of either pharmacological or non-pharmacological alternatives to offer. Interestingly, all patients interviewed by McCorrie et al.^20^ stated that they had experienced adverse side effects from opioids with minimal or no pain relief. There was still concern and apprehension expressed around dose reduction or stopping opioids and the lack of alternatives contributed to this.^20^

#### Theme 2: Relationships in primary care (barrier)

##### Patient expectations

Clinicians found it difficult to engage patients in self-management, with unwillingness to try an opioid reduction resulting in pressure for the prescriber to continue.^21,22^ There was a lack of understanding of the problems with opioid prescribing and therefore management of patient expectations was challenging.

Studies acknowledged that building patient-clinician relationships takes time, skill and effort to achieve.^21,22^ Relationships are impacted by the number of encounters with the same clinician allowing for professional relations to form. This links back to the subtheme of service challenges identified in theme 1. Clinicians felt that the continuation of opioids maintained their patients trust, allowing for non-pharmacological measures to be employed later.^20^ This was echoed by Gill et al.^22^ where clinicians feared breakdown of relations may occur through discussing potential changes. Moreover, McCorrie et al.^20^ expressed that establishing trust is challenging when experience and encounters have resulted in a fixed negative attitude towards a patient.

All three papers cover various geographical areas, including Northern Ireland, rural Wales, East Midlands and the northeast of England. Patient expectation on the limitations of opioid use in CNCP across the United Kingdom is lacking and requires patient education.

##### Liability

Clinicians expressed concerns about professional scrutiny following changes to inappropriate opioid medication where there was a lack of agreement or understanding from patients.^19^ Fear of not meeting patient expectations may lead to formal complaints.^22^ These studies were carried out 15 years apart, and clinician concern of liability is still present.

Seamark et al.^18^ highlighted that clinicians inherit patients discharged from other sectors, which results in primary care clinicians taking over prescribing and therefore responsibility of potentially inappropriate doses. Subsequent changes of opioids can prove challenging as patients may be unwilling.^18^

In addition to professional scrutiny, concerns on the nature of opioid medications and the potential for secondary gains. This includes the sale of prescribed opioid medicines in the community.^18,22^

Three papers; Seamark et al.,^18^ Blake et al. (2),^23^ and Gill et al.^22^ gathered data from different parts of the UK including Cornwall, Nottinghamshire and North Wales, together demonstrating a common theme of liability concerns.

#### Theme 3: Attitudes towards CNCP (barrier)

##### CNCP is difficult to manage

Some GPs expressed discomfort and lack confidence when prescribing opioids for CNCP in practice due to the subjectivity of the condition.^19,20^ The difficulty in managing CNCP occurs particularly when the condition is multi-factorial, and this can pose challenges in clinical judgement and decision-making.^18^ Additionally, raising concerns and initiating a conversation on opioid deprescribing with patients is also a challenge.^18^ There was evidence of clinicians understanding the clinical benefits of opioids in CNCP versus the side effects.^18,20,22^ However, the ability to apply this knowledge in practice with patients still proves to be challenging. These studies were carried out across different localities cross the UK and are inclusive of both doctors and ANP clinicians, thus representing the primary care settings.

##### Fear of opioids versus fear of pain

Explanation of the cause of CNCP could not always be provided which added to patient concerns, further highlighting the complexity of CNCP as a health condition.^19,20^ Patients were aware of and feared dependence towards opioids and aimed to balance pain relief with the adverse effects of opioids.^20^ McCorrie et al.^20^ is the only study that highlights patients’ awareness and understanding of opioid dependence and risks of long-term prescribing. This study was carried out in the north of the country where prescribing rates of opioids are more prevalent. Patients also expressed fear that decreasing their opioid dose would exacerbate their pain.^22^ This links back to theme 1 subtheme; lack of alternatives that patients can be offered when discussing deprescribing.

#### Theme 4: Multidisciplinary team set up (enabler)

##### Clinical pharmacist support

Clinicians expressed positive experiences of working with a clinical pharmacist as part of the MDT to manage opioid prescribing.^21,22^ Gill et al.^22^ and McCann et al.^21^ acquire information and expressions from two professionals (GPs and ANPs) of the MDT observed in the real-world general practice setting. Both studies were carried out within the last 2 years and therefore reflect some of the current changes made to support a wider MDT in primary care.

## Discussion

General practice in the UK is one of the first points of care. This meta-synthesis reviews the barriers and enablers of opioid management indicated for CNCP from the perspective of clinicians and patients.

The key enabler identified articulates that clinicians consider the MDT positive when supporting the management of opioids for CNCP. Clinical pharmacists are highlighted as adding value to the MDT by both doctors and ANPs, bringing expert knowledge of medicines to support with inappropriate prescribing and patient safety. At the time of writing there is no specific evidence evaluating the experiences of clinical pharmacists on managing opioid for CNCP in the UK and this requires further research. The PCN Direct Enhanced Service Contract 25/26 identifies that a variety of AHPs support transformation and service development.^25^ The expansion of these roles within the PCN are reimbursed via the Additional Roles Reimbursement Scheme (ARRS).^26,27^

Unlike previous reviews, this analysis reinforces the current literature and combines experiences from service users (patients) and providers (doctors and ANPs) within general practice in the UK. Many barriers and enablers highlighted align with those identified internationally, for example Australia^28^ and Canada,^29^ supporting global understanding of opioid management.

Despite a rigorous search process some studies may have been missed. The data is limited to a small number of studies and populations, only six studies met the inclusion criteria. Title and abstract screening were carried out by a single reviewer and were not duplicated. Due to working in the general practice setting as a clinical pharmacist, there is an acknowledged potential bias in interpretation.

### Recommendations for policy

The NHS Long-Term Plan outlines the redesign of patient care and addresses health inequalities within in a growing ageing population.^12^ The plan outlines primary care pathways to relieve the pressure on secondary care services. In addition, financial incentives are in place for primary care and PCNs; including Impact and Investment Fund (IIF) and Quality Outcomes Framework (QOF).^25^ In March 2023, NHS England published the ‘Optimising personalised care for adults prescribed medicines associated with dependence or withdrawal symptoms framework’, which addresses the complexity of these classes of medications and outlines a plan for system engagement.^30^ Future policies need to focus on reducing the long waiting times observed for specialist pain services, which support the reduction of inappropriate ongoing opioid prescribing observed in general practice. Opportunities to support non-pharmacological management should be developed to provide alternative options when deprescribing opioids.

### Recommendations for practice

Alignment of both policy and practice is required. This would ensure the best outcomes can be achieved and patient safety optimised. To overcome the barriers, education in multiple areas of the wider NHS network is required. To support with managing patient expectations, patients should be educated on the limitations of opioids indicated for CNCP. Clinicians are aware of the problems associated with inappropriate opioid prescribing. However, clinicians require education and training on soft skills (communication and shared decision-making) to support application of knowledge into consultations.

PCNs should optimise MDTs by utilising suitably trained AHP led clinics for CNCP. Using longer appointment times allocated to ARRS-funded professionals supports with medicines optimisation for CNCP. In addition, both clinicians and patients need to accept a change in culture within the general practice setting.

### Recommendations for research

Current research focuses on doctor professionals and their experiences on opioids for CNCP. Following the proposed recommendations further research is required to identify potential barriers and enablers of additional AHPs managing opioids for CNCP within general practice. For example, at the time of writing there is no current literature on the barriers and enablers of CNCP management experienced by clinical pharmacist AHP’s. Research on the impact of roles, including clinical pharmacists, social prescribers and physiotherapists to name a few, would provide further evidence on medicines optimisation opportunities of opioids at practice level. Further reflection on patient experiences would also add value, including the consequences of opioid deprescribing on one’s mental health and well-being. Further research on patient understanding of opioids is needed, which may differ regionally.

## Conclusion

Exploration of both the barriers and enablers towards opioid management in CNCP has enabled an understanding of the lived experience at practice level. A culture change from service providers and users is required to ensure skill mix is utilised optimally. A movement away from the traditional model of opioids being managed by a doctor needs to occur. Further utilisation of the MDT and AHPs within primary care ensures that patients are seen by the most appropriate practitioner for their needs and would support with safer opioid management.

## Appendix

### Appendix 1

Braun et al.^17^ six steps are outlined below.

Phase 1: Familiarising oneself with the dataset by reading the primary papers and making brief notes on any insights. This started the process of understanding and analysing the data. The studies within this systemic review began to undergo a full overview whilst being understood in relation to the study objectives. This phase was carried out multiple times to become familiar with the papers.

Phase 2: Code generation then occurred to allow for identification of meaningful research concepts through a systematic process, to support with answering my research question. This enabled the primary papers to be brought together. In this study, the barriers and enablers from both patients and clinicians were coded. See Appendix 2 – Photograph of coding example, providing a pictorial image of the coding process.

Phase 3: Generating initial themes then occurred by identifying patterns across the coded data. Combining codes that share a core concept supported me to create these themes. See Appendix 3 – generating initial themes, to observe the initial theme breakdown.

Phase 4: Developing and reviewing themes. Themes were captured around the research question and the objectives. Relationships between the themes were then identified.

Phase 5: Refining, defining and naming themes enabled core concepts to be identified, followed by interpretation of how the findings addressed the study objectives. This aimed to address the barriers and enablers of opioid management in primary care for both clinicians and patients, as well as support with making recommendations to optimise opioid practice.

Phase 6: Writing up involved weaving the data to provide a coherent narrative to answer my research question.

However, a non-linear and flexible approach can be sought to support the data analysis process.^17^ For example, to meet the aim and objectives, the identified papers were read and re-read multiple times to identify barriers, enablers and experiences of opioid management.

### Appendix 3 – generating initial themes

#### Code related to themes

(Seamark et al.^18^) – **C**.

(Blake et al.^19^ (1)) – **D**.

(McCorrie et al.^20^) – **E**.

(McCann et al.^21^) – **L**.

(Blake et al.^23^ (2)) – **P**.

(Gill et al.^22^) – **CC**.

#### Patients

##### Attitudes towards opioids

• Balance of pain control versus side effects of opioids, for example, dependence, constipation and sedation (E).
• Patients seeking pain relief to improve and maintain QOL. Cannot cope with pain anymore therefore time to seek help/obtain appointment to get support (D).
• Fear that decreasing the dose will exacerbate pain (CC).

##### No alternative

• There is a lack of alternative options offered (E).

##### Relationships

• Cannot tell the GP what is troubling them as do not want to waste their time (D).
• GP does not have time to address concerns/time to listen (D).

##### System

• GP is difficult to access (D).
• Lack of continuity of care (E) so patient needs could not be met properly, that is, being given a GP who does not know their history means GP could not meet their needs properly.

#### Clinicians

##### No alternatives

• Lack of new ideas on how to manage pain (E).
• Long-term prescribing of opioids due to lack of alternatives (E).
• Lack of alternatives (CC).
• Lack of alternative and tapering could result in illicit drug use (L).

##### Worried about side effects

• Concerns around side effects including addiction, dependence, sedation, confusion and impaired thinking. Concerns about medical divergence (P, CC).
• Concern about long-term commitment to prescribing, that is, addiction (CC).
• Concerns about the benefits of opioids versus side effects (C).
• Concerns about secondary gains, for example, selling (C).

##### Liability

• Worried about professional scrutiny (P).
• Fear of unmet patient expectations leading to formal complaints (CC).

##### Patient expectation

• Patients pressure towards prescriber (CC).
• Patient expectations and understanding is lacking (CC).
• Lack of a shared management plan (E).
• Patients have their own agenda (E).
• Patient not always willing (L).
• Patients can refuse reduction (CC).
• More patient education is needed on the limitations of opioids (CC).

##### CNCP is hard to diagnose and manage

• Some GPs have discomfort in addressing pain (E).
• Some have a lack of confidence around opioids/pain management (P).
• CNCP is difficult to manage (C).
• Pain is subjective – it’s what the patients day – so difficult to judge (C).
• Challenging to initiate the conversation on opioid deprescribing (L).

##### Relationship with patients

• Negotiation is required that is challenging and managing patient expectations, and challenges engaging patient in self-management (L).
• Establishing trust is challenging when past experience has resulted in a fixed negative attitude towards a patient – that is, GP anticipated that the patient will be a challenge (E).
• Continue opioids maintained trust and will allow non-pharmacological measures to be employed later on (E).
• Building relationships takes time, skill and effort – mutual trust required (CC).
• Breakdown of relations can occur – fear of discussing opioids (CC).

##### System

• Inherited patients discharged on drugs from secondary care (CC).
